# Differential associations of pathogen-specific co-infections with disease severity in pediatric RSV hospitalizations: a 3-year multicenter analysis

**DOI:** 10.1128/spectrum.00945-26

**Published:** 2026-06-15

**Authors:** Dong Xu, Zongming Yang, Xiuyun Zhou, Wankai Xue, Wenjing Zhang, Rui Shi, Yabin Wu, Yanli Wang, Zenghao Xu, Xiaoping Luo, Yongjian Huang, Sainan Shu

**Affiliations:** 1 Department of Pediatrics, Tongji Hospital, Tongji Medical College, Huazhong University of Science and Technology 12443, Wuhan, China; 2 Hubei Provincial Key Laboratory of Pediatric Genetic Metabolic and Endocrine Rare Diseases, Wuhan, China; 3 Hubei Provincial Clinical Research Center for Children’s Growth and Development and Metabolic Diseases, Wuhan, China; 4 Department of Pediatric Pulmonology, Maternal and Child Health Hospital of Hubei Province 477167 , Wuhan, China; 5 Department of Respiratory, Wuhan Children's Hospital, Tongji Medical College, Huazhong University of Science and Technology 12443, Wuhan, Hubei, China; 6 Zheiang Key Lab of Vaccine, Infectious Disease Prevention and Control, Zhejiang Provincial Center for Disease Control and Prevention, Hangzhou, China; Children's National Hospital, George Washington University, Washington, DC, USA

**Keywords:** respiratory syncytial virus, co-infection, disease severity, interaction, multicenter study

## Abstract

Respiratory syncytial virus (RSV) is a leading cause of acute respiratory tract infections (ARTI) in infants and young children. Co-infection with other respiratory pathogens is common in RSV infection. However, the associations of co-infection with disease severity and their potential interactions remain poorly understood, especially in light of evolving epidemiological patterns of respiratory pathogens following the COVID-19 pandemic. This multicenter, retrospective study included 2,862 children under 5 years of age and hospitalized with RSV at three pediatric centers in Wuhan, China, between June 2020 and May 2023. We evaluated the associations of co-infecting pathogens, including bacteria, viruses, fungi, and atypical bacteria, with disease severity, length of hospital stay, and inpatient medical expenses. We found that nearly 44% of children with RSV were co-infected with at least one additional pathogen. Co-infection rates fluctuated in correlation with the implementation and relaxation of COVID-19 non-pharmaceutical interventions. While viral and atypical bacterial co-infections did not significantly alter outcomes, bacterial (OR = 2.00, 95% CI: 1.48, 2.70) and fungal (OR = 2.57, 95% CI: 1.23, 5.35) co-infections were strongly associated with increased disease severity. Both types were also linked to longer hospital stays (days, β = 0.63, 95% CI: 0.38, 0.88 for bacterial; β = 2.68, 95% CI: 1.94, 3.43 for fungal) and higher inpatient medical expenses (Chinese Yuan [CNY], β = 2,317.16, 95% CI: 1,534.43, 3,099.88 for bacterial; β = 6,636.55, 95% CI: 4,366, 8,907.10 for fungal). No significant additive interactions were found between bacterial and fungal co-infections. These findings highlight the importance of distinguishing co-infection types in the clinical management of RSV.

**IMPORTANCE:**

This study clarifies the clinical significance of pathogen co-infections in children hospitalized with respiratory syncytial virus (RSV). We demonstrate a critical distinction that co-infections with bacteria or fungi are associated with more severe disease, longer hospitalizations, and higher costs, whereas viral or atypical bacterial co-infections are not. In the context of evolving RSV epidemiology and pathogen co-infections in the post-pandemic era, this finding may help risk-based clinical management when co-infection exists.

## INTRODUCTION

Respiratory syncytial virus (RSV) is the most common cause of acute respiratory tract infections (ARTI) and a major driver of hospitalization and morbidity in infants and young children worldwide (1, 2). RSV infection presents with a broad spectrum of manifestations, from mild upper respiratory illness to severe lower respiratory tract disease that may need intensive care and can be fatal (3). This heterogeneity suggests that factors beyond the virus itself, such as host characteristics, seasonality, and co-circulating respiratory pathogens, may significantly influence disease outcomes (4, 5).

Molecular detection technologies, including multiplex polymerase chain reaction (PCR), isothermal nucleic acid amplification tests, and next-generation sequencing, have demonstrated that over one-third of children hospitalized with RSV have co-infections with other respiratory pathogens (6, 7). While RSV is typically seasonal with epidemics usually occurring in the winter months, the COVID-19 pandemic and associated non-pharmaceutical interventions (NPIs) have substantially altered its epidemiology and other respiratory virus co-circulation patterns (8–10). However, comprehensive data on how co-infection profiles have changed in children with RSV infection during and after the pandemic remained limited. Moreover, these co-infections may potentially interact with RSV, possibly leading to altered immune responses and more severe clinical courses (11, 12). However, existing evidence on the impact of co-infections on disease severity is generally limited, often focusing narrowly on bacterial or viral co-infections, without a systematic assessment of fungal or atypical bacterial infections (13–16). Few studies have systematically and simultaneously evaluated the independent effects of the four major pathogen categories, including bacteria, viruses, fungi, and atypical bacteria (specifically *Mycoplasma pneumoniae* [MP], *Chlamydia pneumoniae* [CP], and *Legionella pneumophila* [LP]), within the same pediatric cohort. It also remains unclear whether different co-infection types interact synergistically or antagonistically to modulate disease severity in children with RSV infection.

To address these knowledge gaps, we conducted a large, multicenter, retrospective study to examine trends in the prevalence of different respiratory co-infection patterns (bacterial, viral, fungal, atypical bacterial) among children under 5 years hospitalized with RSV during and after the COVID-19 pandemic and to evaluate the independent and joint associations of each major co-infection type with the risk of severity outcomes.

## RESULTS

**Fig 1:**
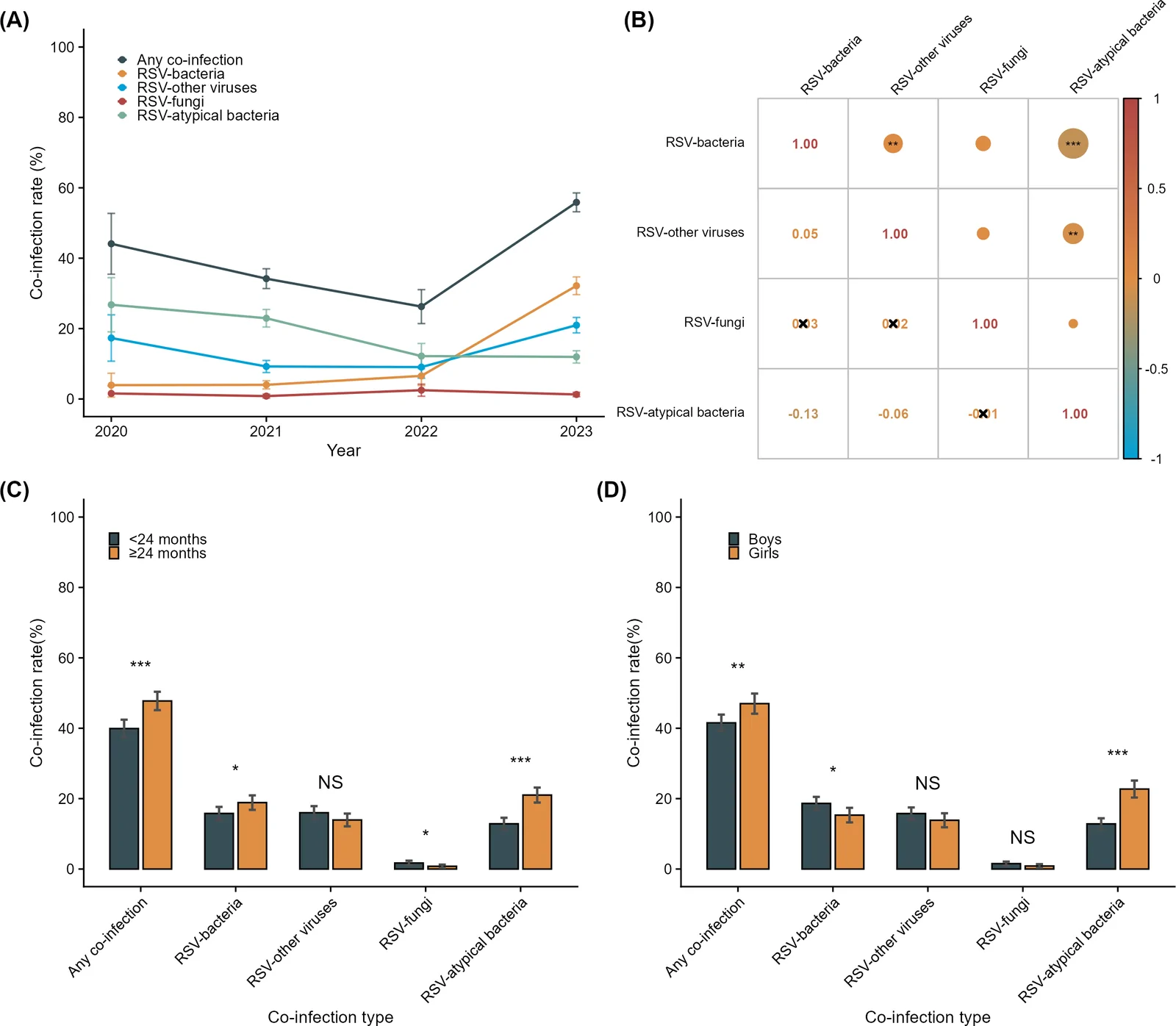
Time trends in co-infection rate (**A**), grade of correlation between different co-infection types (**B**), and comparison of co-infection types by age (**C**) and sex (**D**). NS, not significant; *P < 0.05, **P < 0.01, ***P < 0.001.

A total of 10,684 children hospitalized with ARTI were assessed for eligibility. After excluding 68 cases with data errors or contradictions and 7,754 cases who tested negative for RSV, we included a total of 2,862 children hospitalized with RSV infection (Fig. S1). The median age was 23.00 months (interquartile range, IQR: 8.00, 40.08), and 1,162 (40.6%) were girls. Among the enrolled patients, 1,252 (43.75%) tested positive for at least one additional respiratory pathogen. This included 1,072 cases of double infection (RSV plus one other category of pathogen), 170 cases of triple infection (RSV plus two), and 10 cases of quadruple infection (RSV plus three). The prevalence of RSV-bacterial, RSV-viral, RSV-fungal, and RSV-atypical bacterial co-infections was 495 (17.30%), 429 (14.99%), 36 (1.26%), and 482 (16.84%), respectively. The overall co-infection rate declined during the COVID-19 pandemic but increased sharply after the relaxation of NPIs in late 2022 (Fig. 1A). Correlation analysis revealed generally low associations between different types of RSV-respiratory pathogen co-infections. The strongest negative correlation was observed between RSV-bacterial and RSV-atypical bacterial co-infections (Phi = –0.13, *P* < 0.001; Fig. 1B). The detection rate of any co-infection was significantly higher in children aged ≥24 months compared with those under 24 months of age (47.7% vs 39.9%, *P* < 0.001), and in girls compared with boys (47.0% vs 41.5%, *P* = 0.004) (Fig. 1C and D).

Table 1 summarizes the baseline characteristics of children with RSV infection stratified by co-infection status. Compared with the RSV mono-infection group, children with bacterial or atypical bacterial co-infections tended to be older. Notably, patients with fungal co-infections had a higher prevalence of preterm birth and more severe clinical manifestations at admission than the other groups. The prevalence of comorbidities, duration of symptoms before admission, and proportion of abnormal chest radiographic findings were comparable across all groups.

**TABLE 1:** Baseline characteristics of participant^*a*^^,^^*b*^

	All participants	RSV mono-infection	Double co-infections							
			RSV-bacteria	*P* value	RSV-other viruses	*P* value	RSV-fungi	*P* value	RSV-atypical bacterial	*P* value
Number of participants	2,862	1,610	369		285		16		402	
Age, median (IQR), months	23.00 (8.00, 40.08)	20.07 (6.03, 39.00)	30.82 (9.36, 43.00)	<0.001	18.00 (7.13, 38.88)	0.605	9.53 (4.00, 27.25)	0.324	30.00 (17.00, 43.00)	<0.001
Age groups, *n* (%), months				0.001		0.676		0.168		<0.001
<24	1,463 (51.12)	879 (54.60)	166 (44.99)		160 (56.14)		12 (75.00)		156 (38.81)	
≥24	1,399 (48.88)	731 (45.40)	203 (55.01)		125 (43.86)		4 (25.00)		246 (61.19)	
Sex, *n* (%)				0.956		0.967		>0.999		<0.001
Boys	1,700 (59.40)	994 (61.74)	229 (62.06)		175 (61.40)		10 (62.50)		175 (43.53)	
Girls	1,162 (40.60)	616 (38.26)	140 (37.94)		110 (38.60)		6 (37.50)		227 (56.47)	
Residential area, *n* (%)				<0.001		0.001		0.336		0.444
Wuhan	2,404 (84.00)	1,392 (86.46)	278 (75.34)		225 (78.95)		12 (75.00)		354 (88.06)	
Outside Wuhan	458 (16.00)	218 (13.54)	91 (24.66)		60 (21.05)		4 (25.00)		48 (11.94)	
Preterm birth, *n* (%)	217 (7.58)	108 (6.71)	30 (8.13)	0.393	32 (11.23)	0.01	4 (25.00)	0.017	24 (5.97)	0.673
Comorbidities, *n* (%)										
Any	130 (4.54)	74 (4.60)	17 (4.61)	>0.999	13 (4.56)	>0.999	0 (0.00)	0.783	9 (2.24)	0.047
Respiratory system	20 (0.70)	10 (0.62)	2 (0.54)	>0.999	1 (0.35)	0.896	0 (0.00)	>0.999	1 (0.25)	0.598
Congenital heart disease	53 (1.85)	30 (1.86)	6 (1.63)	0.927	5 (1.75)	>0.999	0 (0.00)	>0.999	6 (1.49)	0.771
Nervous system	11 (0.38)	4 (0.25)	1 (0.27)	>0.999	3 (1.05)	0.125	0 (0.00)	>0.999	0 (0.00)	0.708
Duration of symptoms prior to admission, median (IQR), days	5.00 (4.00, 7.00)	5.00 (4.00, 7.00)	5.00 (3.00, 7.00)	0.200	5.00 (4.00, 8.00)	0.001	4.50 (3.00, 7.25)	0.860	5.00 (4.00, 7.00)	0.250
Observations at admission, *n* (%)										
Poor consciousness/spirit/appetite	116 (4.05)	59 (3.66)	14 (3.79)	<0.001	8 (2.81)	0.032	1 (6.25)	0.861	16 (3.98)	0.704
Wet rales	1,662 (58.07)	917 (56.96)	245 (66.40)	0.001	151 (52.98)	0.237	11 (68.75)	0.487	220 (54.73)	0.453
Wheezing sound	1,073 (37.49)	602 (37.39)	144 (39.02)	0.549	120 (42.11)	0.149	8 (50.00)	0.437	121 (30.10)	0.008
Shortness of breath/three concave signs	289 (10.10)	149 (9.25)	49 (13.28)	0.025	34 (11.93)	0.193	3 (18.75)	0.386	28 (6.97)	0.177
Chest radiological results, *n* (%)				<0.001		0.191		0.142		0.021
Normal	54 (1.89)	36 (2.24)	3 (0.81)		8 (2.81)		0 (0.00)		6 (1.49)	
Bronchitis	863 (30.15)	550 (34.16)	91 (24.66)		77 (27.02)		3 (18.75)		106 (26.37)	
Bronchopneumonia	1,882 (65.76)	1,005 (62.42)	253 (68.56)		195 (68.42)		12 (75.00)		283 (70.40)	
Lobar pneumonia	51 (1.78)	14 (0.87)	17 (4.61)		4 (1.40)		1 (6.25)		6 (1.49)	
Interstitial pneumonia	12 (0.42)	5 (0.31)	5 (1.36)		1 (0.35)		0 (0.00)		1 (0.25)	
Study site, *n* (%)				<0.001		<0.001		0.003		<0.001
Children’s Hospital	570 (19.92)	179 (11.12)	234 (63.41)		75 (26.32)		6 (37.50)		16 (3.98)	
Maternal and Child Health Hospital	615 (21.49)	292 (18.14)	98 (26.56)		79 (27.72)		1 (6.25)		98 (24.38)	
Tongji Hospital	1,677 (58.60)	1,139 (70.75)	37 (10.03)		131 (45.96)		9 (56.25)		288 (71.64)	
Admission year, *n* (%)				<0.001		<0.001		0.347		<0.001
2020	127 (4.44)	71 (4.41)	3 (0.81)		16 (5.61)		1 (6.25)		30 (7.46)	
2021	1,094 (38.23)	720 (44.72)	29 (7.86)		80 (28.07)		4 (25.00)		231 (57.46)	
2022	320 (11.18)	236 (14.66)	14 (3.79)		24 (8.42)		2 (12.50)		33 (8.21)	
2023	1,321 (46.16)	583 (36.21)	323 (87.53)		165 (57.89)		9 (56.25)		108 (26.87)	

^*a*^ RSV, respiratory syncytial virus; IQR, interquartile range; SD, standard deviation; WBC, white blood cell count; NEU, neutrophil count; LYM, lymphocyte count; CRP, C-reactive protein; ALT, alanine aminotransferase; AST, aspartate aminotransferase; Glb, globulin; CNY, Chinese Yuan.

^*b*^ Continuous variables were compared using the Mann-Whitney U test, while categorical variables were analyzed using the χ^2^ or Fisher’s exact test. The RSV mono-infection group was used as the reference for all comparisons.

After adjustment for potential confounders, both RSV-bacterial co-infection (odds ratio [OR] = 2.00, 95% confidence interval [CI]: 1.48, 2.70) and RSV-fungal co-infection (OR = 2.57, 95% CI: 1.23, 5.35) were associated with an increased risk of composite severity outcomes compared to those without these co-infections (Table 2). In contrast, neither RSV-viral (OR = 1.14, 95% CI: 0.86, 1.51) nor RSV-atypical bacterial co-infection (OR = 1.00, 95% CI: 0.75, 1.34) showed a significant association with the composite severity outcome. Similar results were observed for secondary outcomes. Specifically, RSV-bacterial and RSV-fungal co-infections were associated with higher odds of severe infection (OR = 2.34, 95% CI: 1.70, 3.22; and OR = 3.04, 95% CI: 1.43, 6.47), pediatric intensive care unit (PICU) admission (OR = 8.78, 95% CI: 4.99, 15.46; and OR = 5.08, 95% CI: 1.82, 14.21), and oxygen administration (OR = 1.67, 95% CI: 1.21, 2.31; and OR = 2.42, 95% CI: 1.16, 5.07). RSV-bacterial (β = 0.63 days, 95% CI: 0.38, 0.88) and RSV-fungal co-infections (β = 2.68 days, 95% CI: 1.94, 3.43) were also significantly associated with prolonged hospital stays. Higher inpatient medical expenses were observed in cases with RSV-bacterial (β = 2,317.16 CNY, 95% CI: 1,534.43, 3,099.88), RSV-fungal (β = 6,636.55 CNY, 95% CI: 4,366.00, 8,907.10), and RSV-atypical bacterial co-infections (β = 748.12 CNY, 95% CI: 51.62, 1,444.63).

**TABLE 2:** Associations of RSV co-infection patterns with primary and secondary outcomes^*a*^^,^^*b*^

Outcomes	Categories	Number of cases/number at risk	Estimate (95% CI)			
			Model 1	Model 2	Model 3	Model 4
Combined severity outcomes, OR						
RSV-bacteria	No	462/2,367	1.00 (Ref.)	1.00 (Ref.)	1.00 (Ref.)	1.00 (Ref.)
	Yes	131/495	1.68 (1.28, 2.22)	1.94 (1.45, 2.59)	2.01 (1.49, 2.72)	2.00 (1.48, 2.70)
RSV-other viruses	No	498/2,433	1.00 (Ref.)	1.00 (Ref.)	1.00 (Ref.)	1.00 (Ref.)
	Yes	95/429	1.18 (0.91, 1.52)	1.12 (0.86, 1.47)	1.12 (0.85, 1.47)	1.14 (0.86, 1.51)
RSV-fungi	No	575/2,826	1.00 (Ref.)	1.00 (Ref.)	1.00 (Ref.)	1.00 (Ref.)
	Yes	18/36	3.38 (1.73, 6.59)	2.92 (1.45, 5.87)	2.74 (1.33, 5.63)	2.57 (1.23, 5.35)
RSV-atypical bacteria	No	518/2,380	1.00 (Ref.)	1.00 (Ref.)	1.00 (Ref.)	1.00 (Ref.)
	Yes	75/482	0.7 (0.53, 0.92)	0.97 (0.73, 1.29)	0.97 (0.73, 1.30)	1.00 (0.75, 1.34)
Diagnosis of severe infection, OR						
RSV-bacteria	No	350/2,367	1.00 (Ref.)	1.00 (Ref.)	1.00 (Ref.)	1.00 (Ref.)
	Yes	126/495	1.85 (1.39, 2.47)	2.21 (1.63, 2.99)	2.36 (1.72, 3.24)	2.34 (1.70, 3.22)
RSV-other virus	No	394/2,433	1.00 (Ref.)	1.00 (Ref.)	1.00 (Ref.)	1.00 (Ref.)
	Yes	82/429	1.22 (0.93, 1.60)	1.15 (0.86, 1.54)	1.16 (0.86, 1.57)	1.2 (0.89, 1.63)
RSV-fungi	No	459/2,826	1.00 (Ref.)	1.00 (Ref.)	1.00 (Ref.)	1.00 (Ref.)
	Yes	17/36	4.06 (2.06, 7.97)	3.51 (1.71, 7.19)	3.3 (1.57, 6.93)	3.04 (1.43, 6.47)
RSV-atypical bacteria	No	422/2,380	1.00 (Ref.)	1.00 (Ref.)	1.00 (Ref.)	1.00 (Ref.)
	Yes	54/482	0.67 (0.49, 0.92)	0.98 (0.71, 1.36)	0.99 (0.71, 1.37)	1.02 (0.73, 1.43)
PICU admission, OR						
RSV-bacteria	No	45/2,367	1.00 (Ref.)	1.00 (Ref.)	1.00 (Ref.)	1.00 (Ref.)
	Yes	46/495	7.69 (4.43, 13.35)	8.79 (5.03, 15.36)	9.66 (5.45, 17.12)	8.78 (4.99, 15.46)
RSV-other virus	No	71/2,433	1.00 (Ref.)	1.00 (Ref.)	1.00 (Ref.)	1.00 (Ref.)
	Yes	20/429	1.56 (0.93, 2.60)	1.48 (0.87, 2.51)	1.48 (0.85, 2.58)	1.54 (0.86, 2.76)
RSV-fungi	No	83/2,826	1.00 (Ref.)	1.00 (Ref.)	1.00 (Ref.)	1.00 (Ref.)
	Yes	8/36	8.45 (3.69, 19.33)	6.97 (2.94, 16.52)	6.71 (2.65, 17.01)	5.08 (1.82, 14.21)
RSV-atypical bacteria	No	80/2,380	1.00 (Ref.)	1.00 (Ref.)	1.00 (Ref.)	1.00 (Ref.)
	Yes	11/482	0.82 (0.43, 1.57)	1.28 (0.65, 2.5)	1.35 (0.68, 2.67)	1.57 (0.77, 3.21)
Oxygen administration, OR						
RSV-bacteria	No	382/2,367	1.00 (Ref.)	1.00 (Ref.)	1.00 (Ref.)	1.00 (Ref.)
	Yes	99/495	1.51 (1.12, 2.04)	1.69 (1.24, 2.31)	1.70 (1.23, 2.35)	1.67 (1.21, 2.31)
RSV-other virus	No	407/2,433	1.00 (Ref.)	1.00 (Ref.)	1.00 (Ref.)	1.00 (Ref.)
	Yes	74/429	1.12 (0.84, 1.48)	1.07 (0.8, 1.43)	1.05 (0.78, 1.42)	1.07 (0.79, 1.44)
RSV-fungi	No	466/2,826	1.00 (Ref.)	1.00 (Ref.)	1.00 (Ref.)	1.00 (Ref.)
	Yes	15/36	3.12 (1.59, 6.13)	2.71 (1.34, 5.47)	2.56 (1.24, 5.31)	2.42 (1.16, 5.07)
RSV-atypical bacteria	No	419/2,380	1.00 (Ref.)	1.00 (Ref.)	1.00 (Ref.)	1.00 (Ref.)
	Yes	62/482	0.72 (0.53, 0.96)	0.96 (0.70, 1.30)	0.96 (0.70, 1.31)	0.98 (0.71, 1.33)
Length of stay, days, β						
RSV-bacteria	No	NA	0.00 (Ref.)	0.00 (Ref.)	0.00 (Ref.)	0.00 (Ref.)
	Yes	NA	0.55 (0.30, 0.80)	0.62 (0.37, 0.87)	0.65 (0.39, 0.9)	0.63 (0.38, 0.88)
RSV-other virus	No	NA	0.00 (Ref.)	0.00 (Ref.)	0.00 (Ref.)	0.00 (Ref.)
	Yes	NA	0.25 (0, 0.49)	0.23 (−0.01, 0.46)	0.17 (−0.07, 0.4)	0.19 (−0.05, 0.42)
RSV-fungi	No	NA	0.00 (Ref.)	0.00 (Ref.)	0.00 (Ref.)	0.00 (Ref.)
	Yes	NA	3.16 (2.39, 3.93)	3.01 (2.25, 3.77)	2.77 (2.03, 3.52)	2.68 (1.94, 3.43)
RSV-atypical bacteria	No	NA	0.00 (Ref.)	0.00 (Ref.)	0.00 (Ref.)	0.00 (Ref.)
	Yes	NA	−0.05 (−0.29, 0.18)	0.08 (−0.15, 0.32)	0.11 (−0.12, 0.34)	0.14 (−0.09, 0.37)
Inpatient medical expenses, CNY, β						
RSV-bacteria	No	NA	0.00 (Ref.)	0.00 (Ref.)	0.00 (Ref.)	0.00 (Ref.)
	Yes	NA	1,996.83 (1,218.33, 2,775.34)	2,207.02 (1,434.55, 2,979.48)	2,343.81 (1,558.47, 3,129.15)	2,317.16 (1,534.43, 3,099.88)
RSV-other virus	No	NA	0.00 (Ref.)	0.00 (Ref.)	0.00 (Ref.)	0.00 (Ref.)
	Yes	NA	376.35 (−358.68, 1,111.39)	325.02 (−402.95, 1,052.99)	239.97 (−489.54, 969.49)	317.55 (−404.29, 1,039.39)
RSV-fungi	No	NA	0.00 (Ref.)	0.00 (Ref.)	0.00 (Ref.)	0.00 (Ref.)
	Yes	NA	7,829.9 (5,513.96, 10,145.84)	7,458.5 (5,161.75, 9,755.25)	6,908.21 (4,625.69, 9,190.72)	6,636.55 (4,366, 8,907.10)
RSV-atypical bacteria	No	NA	0.00 (Ref.)	0.00 (Ref.)	0.00 (Ref.)	0.00 (Ref.)
	Yes	NA	240.52 (−462.89, 943.92)	625.61 (−80.94, 1,332.15)	647.4 (−55.95, 1,350.76)	748.12 (51.62, 1,444.63)

^*a*^ RSV, respiratory syncytial virus; OR, odds ratio; CI, confidence interval; NA, not applicable; Ref., reference.

^*b*^ Mixed-effect logistic regression models were used to estimate odds ratio, and linear mixed models were used to estimate β. Model 1 was unadjusted, model 2 was adjusted for age and sex, model 3 was further adjusted for history of preterm birth, comorbidities, duration of symptoms prior to admission, residential area, and admission year, and model 4 was further adjusted for other co-infections.

Children with both bacterial and fungal co-infections had elevated risks of severe infection (OR = 9.39, 95% CI: 1.76, 50.12) and PICU admission (OR = 40.61, 95% CI: 5.33, 309.53) compared to those with neither co-infection (Table 3). However, no significant additive interaction was detected between RSV-bacterial and RSV-fungal co-infections for any of the severity outcomes. The relative excess risk due to interaction (RERI) was 3.20 (95% CI: –2.71, 9.11) for the composite outcome, 4.38 (95% CI: –2.73, 11.49) for severe infection, 26.00 (95% CI: –25.66, 77.66) for PICU admission, and 1.82 (95% CI: –3.69, 7.33) for oxygen administration.

**TABLE 3:** Joint associations of RSV-bacterial and RSV-fungal co-infections with primary and secondary outcomes^*a*^^,*b*^

Subgroup	Number of cases/number at risk	OR (95% CI)	RERI (95% CI)
Combined severity outcomes			
No RSV-bacterial co-infection			
No RSV-fungal co-infection	451/2,341	1.00 (Ref.)	NA
RSV-fungal co-infection	11/26	2.35 (1.00, 5.55)	NA
RSV-bacterial co-infection			
No RSV-fungal co-infection	124/485	1.97 (1.45, 2.68)	NA
RSV-fungal co-infection	7/10	6.52 (0.92, 46.36)	3.20 (−2.71, 9.11)
Diagnosis of severe infection			
No RSV-bacterial co-infection			
No RSV-fungal co-infection	340/2,341	1.00 (Ref.)	NA
RSV-fungal co-infection	10/26	2.71 (1.12, 6.58)	NA
RSV-bacterial co-infection			
No RSV-fungal co-infection	119/485	2.30 (1.66, 3.19)	NA
RSV-fungal co-infection	7/10	9.39 (1.76, 50.12)	4.38 (−2.73, 11.49)
PICU admission			
No RSV-bacterial co-infection			
No RSV-fungal co-infection	42/2,341	1.00 (Ref.)	NA
RSV-fungal co-infection	3/26	4.90 (1.30, 18.44)	NA
RSV-bacterial co-infection			
No RSV-fungal co-infection	41/485	8.72 (4.82, 15.76)	NA
RSV-fungal co-infection	5/10	40.61 (5.33, 309.53)	26.00 (−25.66, 77.66)
Oxygen administration			
No RSV-bacterial co-infection			
No RSV-fungal co-infection	373/2,341	1.00 (Ref.)	NA
RSV-fungal co-infection	9/26	2.15 (0.89, 5.20)	NA
RSV-bacterial co-infection			
No RSV-fungal co-infection	93/485	1.64 (1.18, 2.28)	NA
RSV-fungal co-infection	6/10	4.61 (0.94, 22.64)	1.82 (−3.69, 7.33)

^*a*^ RSV, respiratory syncytial virus; OR, odds ratio; CI, confidence interval; Ref., reference; RERI, relative excess risk due to interaction; NA, not applicable.

^*b*^ Mixed-effect logistic regression models were used to estimate OR and RERI. The models were adjusted for age, sex, history of preterm birth, comorbidities, duration of symptoms prior to admission, residential area, admission year, RSV-viral co-infection, and RSV-atypical bacterial co-infection.

**Fig 2:**
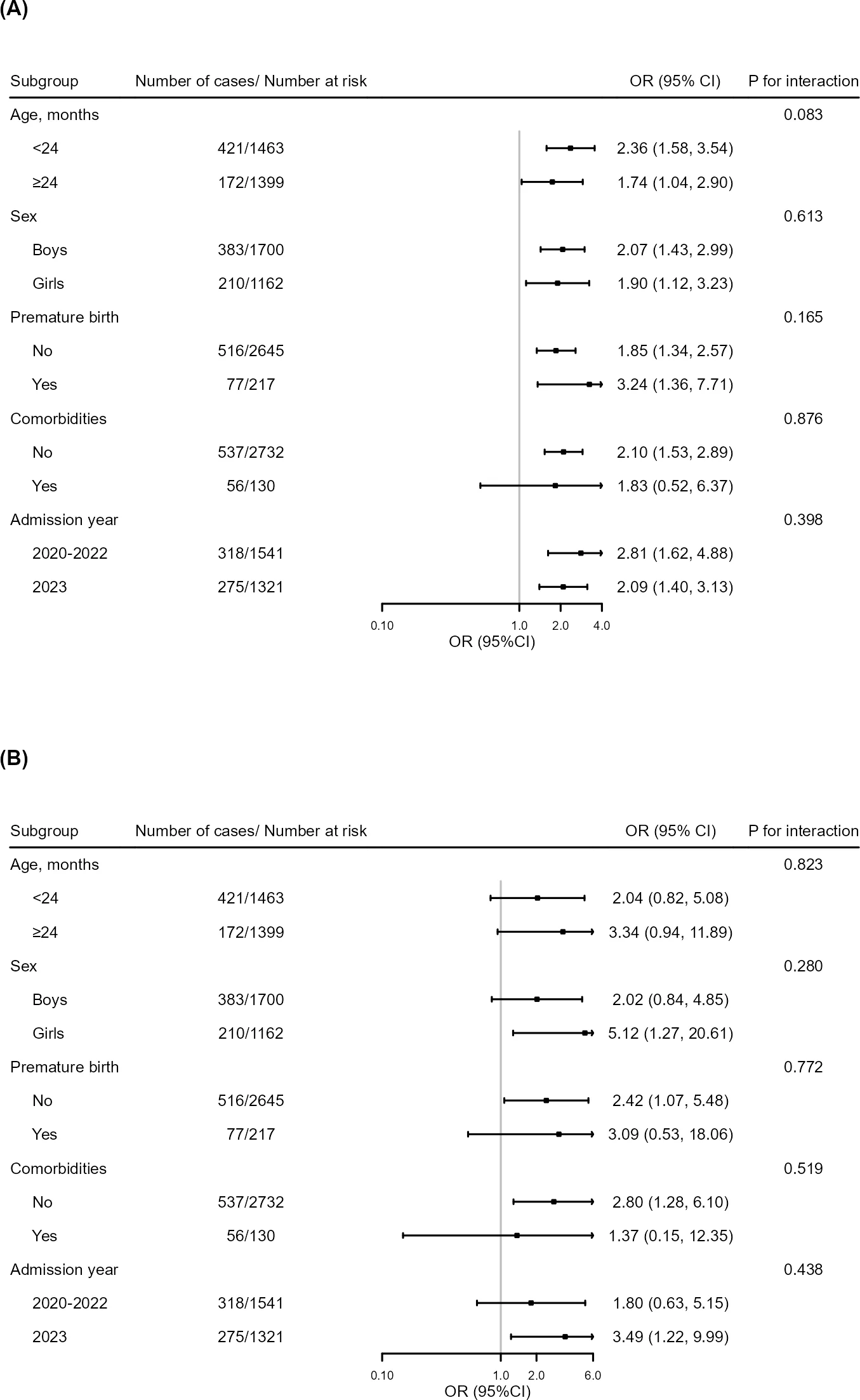
Forest plot for the associations of RSV-bacterial (**A**) and RSV-fungal (**B**) co-infections with primary outcome in subgroups. OR, odds ratio; CI, confidence interval. Mixed-effect logistic regression models were used to estimate OR and were adjusted for age, sex, history of preterm birth, comorbidities, duration of symptoms prior to admission, residential area, admission year, and other co-infections. To calculate *P* values for interaction, likelihood ratio tests were conducted by comparing the maximum likelihood χ² values from nested models with and without the multiplicative interaction term between RSV-bacterial or RSV-fungal co-infections and stratification factors. Specifically for panel B, given the limited sample size (*n* = 36) in the fungal co-infection group and the wide CIs, these results are considered preliminary and should be interpreted with caution.

The associations of RSV-bacterial (Fig. 2A) and RSV-fungal co-infections (Fig. 2B) with the primary outcome were consistent across all subgroups (all *P*-interaction > 0.05). Sensitivity analyses reaffirmed the association between RSV-bacterial co-infection and the primary outcome (Table S3). However, after excluding children with triple or quadruple co-infections (OR = 1.82, 95% CI: 0.61, 5.49) or adjusting for testing items in the sub-cohort analysis (OR = 2.41; 95% CI: 0.86, 6.61), the association for RSV-fungal co-infection was attenuated and became non-significant, likely due to the reduced sample size.

## DISCUSSION

In this multicenter retrospective study of 2,862 children under 5 years hospitalized with RSV, we found that co-infection with other respiratory pathogens was common, affecting 43.75% of the cohort. A significant shift in co-infection rate surrounding the COVID-19 pandemic was observed, with a decline during the period of strict NPIs, followed by a rapid resurgence after their relaxation. We also found that both bacterial and fungal co-infections were independently associated with increased risks of severe outcomes, prolonged hospitalization, and higher inpatient medical expenses. However, no such associations were observed for co-infections with other viruses or atypical bacteria. Furthermore, despite the markedly elevated risk observed in children with both bacterial and fungal co-infections, we did not detect an additive interaction between these two co-infection types on disease severity.

Our study represents one of the few to simultaneously evaluate the associations between four major categories of respiratory pathogen co-infections and clinical outcomes in children hospitalized with RSV. While previous studies have documented the individual impacts of bacterial or viral co-infections, our comprehensive approach provides a broader perspective (13, 14, 17–19). The observed association between bacterial co-infection and increased RSV severity is consistent with a substantial body of literature (6, 13, 17). In contrast, evidence regarding the impact of viral co-infections on disease severity remains conflicting. For instance, a systematic review and meta-analysis reported no significant difference in clinical severity between RSV mono-infection and viral co-infection, except in cases involving human metapneumovirus (19). Conversely, a prospective Belgian study found that children with RSV mono-infection had higher severity scores than those with viral co-infections (18). More recently, a large US population-based surveillance study reported that viral co-detection was not associated with worse outcomes compared to RSV monodetection; co-detection with parainfluenza virus or adenovirus was even associated with lower odds of hospitalization and oxygen use, respectively (14). Within this context of conflicting evidence, our study provides clarifying evidence by demonstrating that, after adjustment for key confounders, including other co-infections, no significant association was observed between RSV-viral co-infection and an increased risk of severe outcomes. It should be noted, however, that our analysis did not differentiate between specific viral types; the overall absence of association may have been influenced by the most commonly co-infected viruses.

Fungal co-infection was relatively uncommon in our cohort, and its relationship with disease severity has not been well characterized in previous studies. Among the limited available literature, only one single-center study reported an association between a higher abundance of Malassezia globosa and a lower risk of hospitalization among infants with RSV infection (20). Our findings demonstrated that RSV-fungal co-infection was associated with the greatest increase in length of stay and inpatient costs, underscoring its clinical relevance. Furthermore, we observed that children with RSV–fungal co-infection tended to be younger, have a history of preterm birth, and present with more severe clinical signs upon admission. These characteristics may provide preliminary evidence to aid in identifying high-risk populations.

Interestingly, RSV-atypical bacterial co-infection was not significantly associated with most severity outcomes, except for a modest increase in inpatient costs. This finding contrasts with a previous, smaller study (*n* = 182) conducted in younger children (<2 years), which reported that those with MP co-infection had a higher prevalence of fever, flaky opacities on chest X-ray, extrapulmonary manifestations, and longer hospital stays compared to those with RSV bronchiolitis alone (21). However, a more recent study suggested that the proportion of severe pneumonia cases and need for bronchoscopic intervention was lower in children with MP and RSV co-infection than in those with MP mono-infection, implying a potential inhibitory interaction (22). Indeed, our data that included both RSV-negative and RSV-positive participants revealed a negative multiplicative interaction between RSV and atypical bacterial infection (β = −0.513, *P* = 0.002, data not shown). Although a definitive explanation based on current data remains challenging, our results suggest that atypical bacterial co-infection may not be associated with significantly elevated severity risk in RSV-infected children. The nature of the interaction between RSV and atypical pathogens warrants further investigation.

Beyond dual infections, multiple co-infections (i.e., RSV with two or more other pathogens) are also clinically relevant, as they may indicate a higher microbial burden and potential for synergistic severity mechanisms (23, 24). In the present study, a substantial proportion of co-infected children (180/1,252, 14.4%) had triple or quadruple co-infections. The present study also assessed the possible additive interactions between RSV-bacterial and RSV-fungal co-infections, which are two types independently associated with severe outcomes. The results revealed that the combined effect of RSV-bacterial and RSV-fungal co-infections was similar to the sum of their individual effects, as indicated by a non-significant RERI. This finding of no additive interaction should, however, be interpreted with caution due to the limited number of children with concurrent RSV-bacterial and RSV-fungal co-infections, which reduced the statistical power to detect a significant interaction effect.

Findings of our study should be interpreted with acknowledgment of several limitations. First, due to its retrospective design, the influence of unmeasured confounding factors, such as specific pathogen subtypes or host genetic factors, cannot be entirely ruled out. Moreover, we could not precisely differentiate between co-infections present at admission and superinfections acquired during hospitalization. Second, variations in routine clinical testing protocols across the participating hospitals may have introduced some detection bias in the classification of co-infections, although we attempted to mitigate this by adjusting for the study site as a random effect and performing sensitivity analyses. Furthermore, because more severely ill children were more likely to undergo comprehensive microbiological testing, detection bias could have inflated the observed associations between bacterial and fungal co-infections and clinical severity. However, our sensitivity analysis showed robust results after adjusting for the diagnostic tests ordered, suggesting that the findings were not merely an artifact of differential testing practices. Third, the relatively small number of fungal co-infection cases limited the statistical power and precision of the associated risk estimates, as suggested by the attenuated association after excluding patients with triple or quadruple co-infections. Future studies with larger sample sizes are needed to confirm the association between fungal co-infection and RSV severity. Finally, as our study population was recruited from three major pediatric centers in a single city, the generalizability of our findings to other regions or healthcare settings requires further validation.

In summary, bacterial and fungal co-infections were independently associated with more severe outcomes in children hospitalized with RSV, whereas viral or atypical bacterial co-infections were not. However, we found no evidence of an additive interaction between concurrent bacterial and fungal co-infections. These findings highlight the importance of distinguishing co-infection types in the clinical management of RSV. Future studies are warranted to elucidate the nature of the interaction between RSV and atypical bacteria and to validate the impact of fungal co-infection in larger cohorts.

## MATERIALS AND METHODS

### Study design and participants

This hospital-based, retrospective study utilized data from three major pediatric medical centers in Wuhan, China, including Tongji Hospital, Wuhan Children’s Hospital, and Maternal and Child Health Hospital of Hubei Province. Participating hospitals provided de-identified data, including diagnostic and procedure codes, billing records, and administrative information. The study included children under 5 years of age who were hospitalized with ARTI between 15 June 2020 and 30 May 2023. We categorized the study duration into two distinct phases based on national health policy: the strict NPIs period (June 2020 to December 2022), characterized by rigorous pandemic control measures, and the relaxation period (January 2023 to May 2023), following the adjustment of COVID-19 management strategies (25). We identified ARTI cases based on the principal discharge ICD-10 codes, with J00–J06 for acute upper respiratory infections, J09–J18 for influenza and pneumonia, and J20–J22 for other acute lower respiratory infections. We focused on ARTI cases with laboratory-confirmed RSV infection.

### Laboratory testing and case definition

RSV infection and co-infecting pathogens were identified using a standardized 13-target multiplex real-time PCR panel conducted on nasopharyngeal swabs, nasal flock swabs, nasopharyngeal aspirates, tracheal aspirates, bronchial aspirates, or bronchoalveolar lavage fluid. This single panel simultaneously screened all patients for 11 viral targets (RSV, rhinovirus, bocavirus, parainfluenza virus, coronavirus, influenza A virus, H1N1, H3N2, metapneumovirus, influenza B virus, and adenovirus) and 2 atypical bacterial targets (MP and CP). Beyond this universal screening, additional microbiological investigations were performed based on each hospital’s clinical guidelines or clinician decisions to capture a broader pathogen spectrum. These included conventional methods, such as smear microscopy, bacterial/fungal/mycobacterial cultures, and (1,3)-β-D-glucan (G) and galactomannan (GM) assays, as well as serological antibody detection for five viruses (parainfluenza viruses, RSV, influenza A virus, influenza B virus, and adenovirus) and three atypical pathogens (MP, CP, and LP). These testing methodologies were applied uniformly across the three hospitals. Patients were classified into the co-infection group if any virus (other than RSV) or atypical bacteria was detected, while for bacterial and fungal detections, co-infection was determined by pediatricians through a comprehensive assessment of the pathogen’s pathogenicity and clinical characteristics to distinguish between clinically significant pathogens and potential colonizers (Table S1). Co-infections were categorized as RSV-bacterial, RSV-other viral, RSV-fungal, or RSV-atypical bacterial. These categories were not mutually exclusive, as some children had RSV detected alongside two or more other pathogens.

### Covariates data extraction

Data were extracted from electronic health records using a standardized case report form. Demographic variables included age (in months), sex (boys, girls), and residential area (in Wuhan, outside Wuhan). Clinical characteristics encompassed history of preterm birth (no, yes), comorbidities (no, yes), duration of symptoms prior to admission (days), observations at admission (consciousness/spirit/appetite, wet rales, wheezing sound, shortness of breath/three concave signs), chest radiological results (normal, bronchitis, bronchopneumonia, lobar pneumonia, interstitial pneumonia), and hospital admission and discharge dates. Missing values for covariates occurred in less than 0.5% of cases (Table S2) and were managed via mode imputation for categorical variables to maintain the sample size for analysis.

### Outcomes

The primary outcome was a composite measure of disease severity, which included diagnosis of severe infection by a pediatrician, PICU admission, or oxygen administration during hospitalization. A diagnosis of severe infection was established if the patient met at least one of the following criteria: poor general condition; refusal to feed or signs of dehydration; impaired consciousness; significantly increased respiratory rate (infants ≥ 70 breaths/min, older children ≥ 50 breaths/min); cyanosis; dyspnea; oxygen saturation <92% at sea level or <90% in high-altitude areas; multi-lobar involvement or >2/3 involvement of a single lobe; pleural effusion; or extrapulmonary complications (26). Secondary outcomes included each component of the primary outcome, length of stay, and inpatient medical expenses.

### Statistics analysis

Baseline characteristics were summarized as median (IQR) for continuous variables, and as numbers (percentages) for categorical variables. Only patients with RSV mono-infection or dual co-infections were included in the baseline description to ensure statistical independence. Data were compared across groups using the Mann-Whitney U test for continuous variables and the χ^2^ test or Fisher’s exact test for categorical variables, with the RSV mono-infection group serving as the reference. We also calculated the annual co-infection rate for each type. Correlations between types of co-infections were assessed using the Phi coefficient (27). Age- and sex-specific co-infection rates were compared using χ^2^ or Fisher’s exact tests.

In primary analysis, we used a generalized linear mixed model with a logit link function, treating study site as random effects, to estimate ORs and 95% CIs of binary outcomes comparing children with specific co-infection patterns to those without. For continuous outcomes, we used an identity link to estimate mean differences. Model 1 was unadjusted, model 2 was adjusted for age and sex, model 3 was further adjusted for history of preterm birth, comorbidities, duration of symptoms prior to admission, residential area, and admission year, and model 4 was further adjusted for other co-infections. Because both RSV-bacterial co-infection and RSV-fungal co-infection were independently associated with increased risk of disease severity, we calculated the ORs of severity outcomes related to combinations of co-infection status and estimated potential additive interaction as the RERI. The 95% CIs of the RERI were generated by drawing 1,000 bootstrap samples from the estimation data set (28). If there were no additive interaction, the CIs of the RERI would include 0.

We conducted subgroup analyses to test for effect modification by including interaction terms in the fully-adjusted model for the following groups: age (<24 months, and ≥24 months), sex (boys, girls), preterm birth (no, yes), comorbidities (no, yes), and admission year (2020–2022, 2023). We compared the maximum likelihood χ^2^ from a nested model with and without the multiplicative interaction term. We also performed several sensitivity analyses to examine the robustness of the results. First, we excluded children with preterm birth or with any comorbidities. Second, we excluded children with symptom duration >7 days prior to admission. Third, we excluded children with triple or quadruple co-infections to minimize residual confounding. Fourth, we adjusted for study site as a confounder, rather than treating it as a random effect (29). Last, to mitigate potential detection bias, we conducted an additional analysis in a sub-cohort from Tongji Hospital (*n* = 1,188) by further adjusting for whether bacterial, fungal, or atypical pathogen tests were performed.

## SUPPLEMENTAL MATERIAL

Supplemental tables and figure spectrum.00945-26-s0001.docx Tables S1 to S3 and Figure S1.

ASM does not own the copyrights to Supplemental Material that may be linked to, or accessed through, an article. The authors have granted ASM a non-exclusive, world-wide license to publish the Supplemental Material files. Please contact the corresponding author directly for reuse.
