# Supplementary material for: Differential associations of pathogen-specific co-infections with disease severity in pediatric RSV hospitalizations: a 3-year multicenter analysis
Source: Microbiol Spectr. 2026 Jun 15;14(7):e00945-26. doi: 10.1128/spectrum.00945-26 (PMC13339963; doi:10.1128/spectrum.00945-26)

**Table S1** Standard comments for detected bacteria and fungi

| **Respiratory tract microbes divided in different categories according to their potential clinical relevance** | | |
| --- | --- | --- |
| **Category** | **Bacteria** | **Fungi** |
| **A: Always pathogens**  Always considered relevant in a patient with respiratory tract infection | *Bordetella pertussis* | **-** |
| **B: Usually pathogens**  Can be colonizers, but are usually considered relevant in a patient with pneumonia | *Haemophilus influenzae*  *Streptococcus pneumoniae* | *Aspergillus* |
| **C: Usually not pathogens**  Usually colonizers, but can cause pneumonia, especially in patients with immunosuppression or underlying conditions | *Staphylococcus aureus*  *Moraxella catarrhalis*  *Klebsiella pneumoniae*  *Escherichia coli*  *Acinetobacter baumannii*  *Pseudomonas aeruginosa* | *Candida* |

**Note:** This table was adapted and modified from *Markussen DL, Serigstad S, Ritz C, et al. Diagnostic Stewardship in Community-Acquired Pneumonia With Syndromic Molecular Testing: A Randomized Clinical Trial.* ***JAMA Netw Open****. 2024 Mar 4;7(3):e240830.* Final determination of co-infection was made by the treating pediatricians, who integrated these microbiological findings with each patient's clinical presentation, inflammatory markers, and radiological evidence to differentiate between true pathogens and potential colonization.

**Table S2** Missing proportions of covariates

| **Variables** | **Number of missing cases** | **Total participants** | **Missing proportion, %** |
| --- | --- | --- | --- |
| Age | 0 | 2862 | 0 |
| Sex | 0 |  | 0 |
| Residential area | 0 |  | 0 |
| Preterm birth | 0 |  | 0 |
| Comorbidities | 0 |  | 0 |
| Duration of symptoms prior to admission | 0 |  | 0 |
| Poor consciousness/spirit/appetite | 0 |  | 0 |
| Wet rales | 0 |  | 0 |
| Wheezing sound | 2 |  | 0.07 |
| Shortness of breath/Three concave sign | 1 |  | 0.03 |
| Chest radiological results | 12 |  | 0.42 |
| Study site | 0 |  | 0 |
| Admission year | 0 |  | 0 |

**Table S3** Sensitivity analyses for the associations of RSV-bacterial and RSV-fungal co-infections with primary outcome

| **Outcomes** | **Categories** | **Number of cases/**  **Number at risk** | **OR (95% CI)** |
| --- | --- | --- | --- |
| **Analysis 1: Excluded children with premature birth or with any comorbidities** | | | |
| RSV-bacteria | No | 379/2118 | **1.00 (Ref.)** |
|  | Yes | 96/425 | 1.94 (1.38, 2.72) |
| RSV-other viruses | No | 407/2179 | **1.00 (Ref.)** |
|  | Yes | 68/364 | 1.1 (0.81, 1.5) |
| RSV-fungi | No | 464/2518 | **1.00 (Ref.)** |
|  | Yes | 11/25 | 2.81 (1.19, 6.62) |
| RSV-atypical bacteria | No | 410/2103 | **1.00 (Ref.)** |
|  | Yes | 65/440 | 1.06 (0.78, 1.44) |
| **Analysis 2: Excluded children with symptom duration > 7 days prior to admission** | | | |
| RSV-bacteria | No | 385/1936 | **1.00 (Ref.)** |
|  | Yes | 104/378 | 2.19 (1.54, 3.11) |
| RSV-other virus | No | 422/2003 | **1.00 (Ref.)** |
|  | Yes | 67/311 | 1.09 (0.78, 1.51) |
| RSV-fungi | No | 475/2289 | **1.00 (Ref.)** |
|  | Yes | 14/25 | 3.01 (1.19, 7.61) |
| RSV-atypical bacteria | No | 424/1929 | **1.00 (Ref.)** |
|  | Yes | 65/385 | 1.15 (0.84, 1.59) |
| **Analysis 3: Excluded children with triple or quadruple co-infections** | | | |
| RSV-bacteria | No | 448/2313 | **1.00 (Ref.)** |
|  | Yes | 86/369 | 1.64 (1.15, 2.34) |
| RSV-other virus | No | 481/2397 | **1.00 (Ref.)** |
|  | Yes | 53/285 | 0.95 (0.67, 1.36) |
| RSV-fungi | No | 528/2666 | **1.00 (Ref.)** |
|  | Yes | 6/16 | 1.82 (0.61, 5.49) |
| RSV-atypical bacteria | No | 482/2280 | **1.00 (Ref.)** |
|  | Yes | 52/402 | 0.82 (0.59, 1.15) |
| **Analysis 4: Adjusted for study site as a confounder** | | | |
| RSV-bacteria | No | 462/2367 | **1.00 (Ref.)** |
|  | Yes | 131/495 | 1.97 (1.46, 2.66) |
| RSV-other virus | No | 498/2433 | **1.00 (Ref.)** |
|  | Yes | 95/429 | 1.18 (0.89, 1.55) |
| RSV-fungi | No | 575/2826 | **1.00 (Ref.)** |
|  | Yes | 18/36 | 2.96 (1.44, 6.07) |
| RSV-atypical bacteria | No | 518/2380 | **1.00 (Ref.)** |
|  | Yes | 75/482 | 1.01 (0.75, 1.34) |
| **Analysis 5: adjusted for specific microbiological tests performed in a sub-cohort from Tongji Hospital** | | | |
| RSV-bacteria | No | 251/1143 | **1.00 (Ref.)** |
|  | Yes | 22/45 | 3.17 (1.59, 6.29) |
| RSV-other virus | No | 242/1077 | **1.00 (Ref.)** |
|  | Yes | 31/111 | 1.27 (0.77, 2.03) |
| RSV-fungi | No | 263/1167 | **1.00 (Ref.)** |
|  | Yes | 10/21 | 2.41 (0.86, 6.61) |
| RSV-atypical bacteria | No | 228/956 | **1.00 (Ref.)** |
|  | Yes | 45/232 | 0.98 (0.66, 1.43) |

**Abbreviations:** RSV=respiratory syncytial virus; OR=odds ratio; CI=confidence interval; Ref.=reference

**Note: (**Mixed effect) logistic regression models were used to estimate odds ratio, with adjustment for age, sex, history of premature birth, comorbidities, duration of symptoms prior to admission, residential area, and admission year, and other co-infections.

**Figure S1** Flowchart of the study design


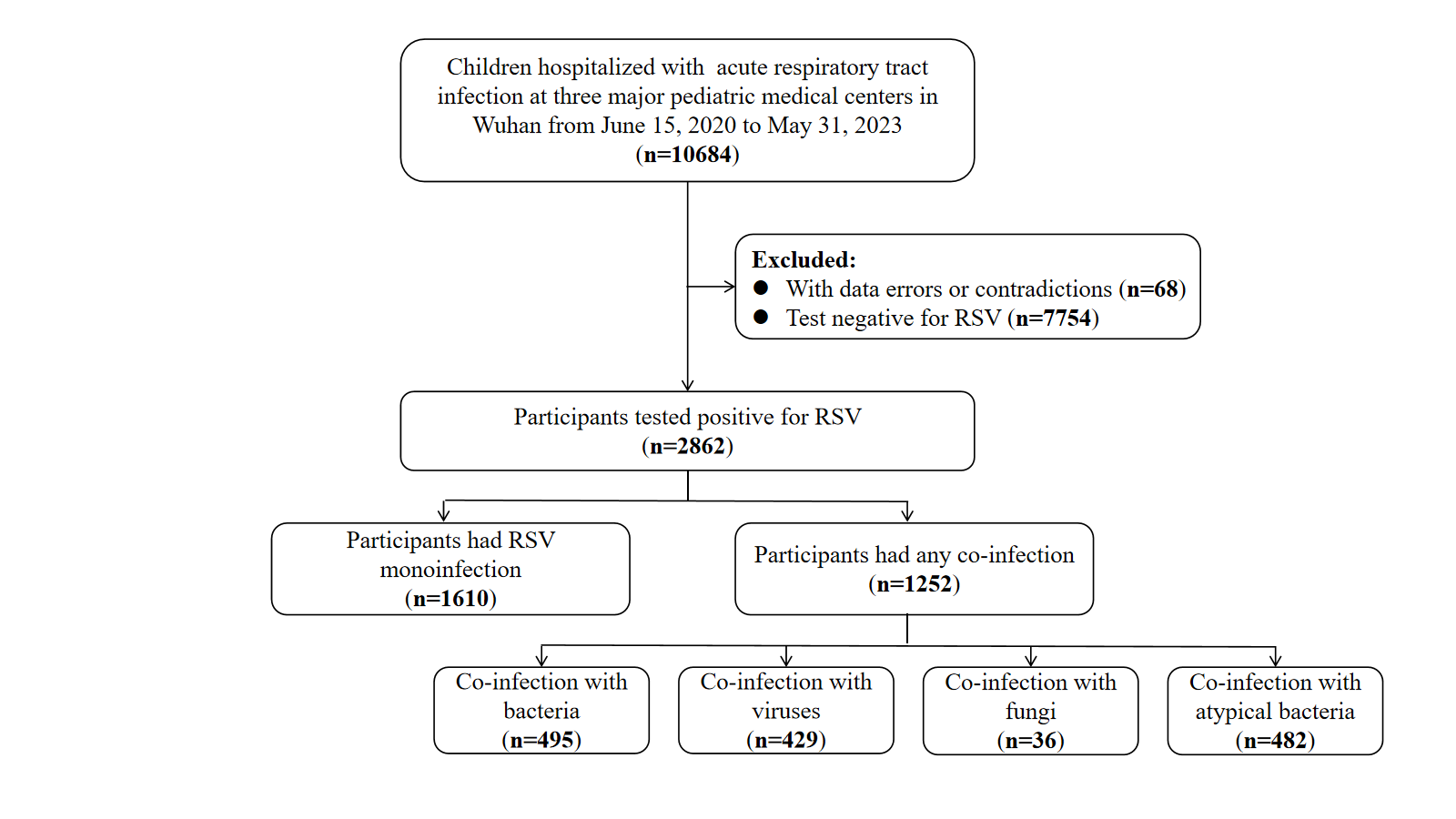

Supplement: Supplemental tables and figure — Tables S1 to S3 and Figure S1. [file spectrum.00945-26-s0001.docx]
